# Simple smoke ventilation method for uniportal video-assisted thoracoscopic surgery

**DOI:** 10.1093/icvts/ivac061

**Published:** 2022-03-03

**Authors:** Yasushi Mizukami, Ryunosuke Maki, Hirofumi Adachi

**Affiliations:** Department of Thoracic Surgery, National Hospital Organization, Hokkaido Cancer Center, Hokkaido, Japan

**Keywords:** Uniportal video-assisted thoracoscopic surgery, Pulmonary resection, Surgical smoke and mist

## Abstract

Fogging of the thoracoscopic lens affects a surgeon’s ability to maintain a clear operating field. In uniportal video-assisted thoracoscopic surgery, the thoracoscopic lens tends to fog when the surgeon does not hold a suction instrument. Thus, a suction instrument needs to be held by the surgeon’s nondominant hand to remove surgical smoke, mist, and moisture. Here, we describe a simple, easy and cost-effective surgical smoke ventilation technique for uniportal video-assisted thoracoscopic surgery using a suction catheter to solve the problem. We present this technique and comment on its advantages, including decreased cost and improved surgical visualization.

## INTRODUCTION

Uniportal video-assisted thoracoscopic surgery (UVATS), which reduces the patient’s duration of hospital stay and postoperative pain, is performed globally [1]. Diego *et al.* reported the use of a long suction cannula, vessel-sealing devices and long graspers to provide an adequate field of view while performing the surgical resection [2]. On the other hand, fogging of the thoracoscopic lens is among the factors that affect a surgeon’s ability to maintain a clear operating field. In conventional multiportal video-assisted thoracoscopic surgery (MVATS), removal of surgical smoke and mist is easy using suction by the assistant surgeon or ventilation from ports. However, in UVATS, because the surgical port is small and spontaneous removal of smoke and mist is difficult, a suction instrument held by the surgeon’s nondominant hand is generally required. The thoracoscopic lens tends to fog when the surgeon does not hold the suction instrument, for example while holding the forceps instead. Another reason for moisture in the thoracic cavity is the small wound. In particular, fogging is remarkable when a 5-mm thoracoscope is used compared with a 10-mm thoracoscope. It has been reported that surgical smoke cannot be ignored as a biohazard [3]. Removal of surgical smoke is important during the COVID-19 epidemic. Here, we describe a simple, easy and cost-effective surgical smoke removal technique for UVATS using a suction catheter. We present this technique and comment on its advantages, including surgical visualization.

## TECHNIQUE

Ethics approval for this study was granted by the ethics committee of Hokkaido Cancer Center on 2 Nov 2021 (approval number: 03-56); the requirement to obtain informed consent directly was waived.

UVATS was performed with the patient in the lateral decubitus position under general anaesthesia. The trachea was canalized with a double-lumen tube for selective ventilation of the lung. A 3- to 4-cm operator’s port was made between the middle axillary line and the anterior axillary line (basically the fourth intercostal space for the upper and middle lobe and the fifth intercostal space for the lower lobe). A protective film and ring device for protecting the wound (Alexis^®^ Wound Protector/Retractor XS; Applied Medical Inc., Rancho Santa Margarita, CA, USA) was used. A rigid 5-mm 30° video thoracoscope was inserted. Antifog solution was not used, and the lens of the video thoracoscope was warmed with hot water and wiped with gauze. A vessel-sealing device or an electrocautery device was introduced into the incision by the surgeon. The temperature of our operating room was controlled by the automatic air-conditioning system and the temperature setting was set to 26°C.

**Figure 1:**
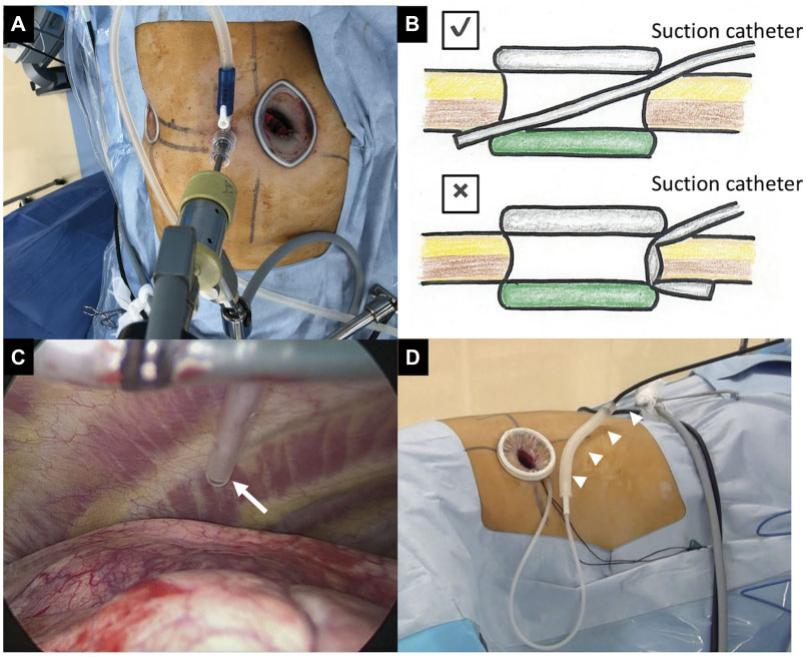
Simple surgical smoke ventilation method. (**A**) How to ventilate mist and smoke during conventional multiportal video-assisted thoracoscopic surgery. The suction tube is connected to the camera port. (**B**) A cross-section schema. The top schema is the correct method. The suction catheter is inserted straight. The suction catheter is kinked in the lower schema. (**C**) The head of the suction catheter is seen from the side of the wound retractor in the thoracic cavity (white arrow). (**D**) When the suction cannula is not used in the surgical field, the suction catheter is connected to the suction cannula via the connection tube (white arrowheads). The suction cannula may be connected to the suction tube directly.

In our conventional MVATS, a camera port connected to a suction tube was used to ventilate surgical smoke and mist to prevent fogging of the thoracoscopic lens (Fig. 1A). In UVATS, connecting a uniport with a suction tube is difficult. Accordingly, in our technique, a sterilized suction catheter was passed along the side of the protective film and ring device. The suction catheter must be inserted straight because it is easy to kink (Fig. 1B and C). When the suction cannula is not used in the surgical field, the suction catheter is connected to the suction cannula via the suction tube (Fig. 1D). Video 1 shows how to attach the suction catheter.

We performed uniportal thoracoscopic lobectomy or segmentectomy and compared cases between May 2021 to August 2021 (before introducing the use of our method) with cases between September 2021 and October 2021 after we applied our novel technique. The number of times the videoscope lens was washed using hot water and wiped intraoperatively was measured and compared. Statistical analysis was performed using JMP^®^ 14 (SAS Institute Inc., Cary, NC, USA). The Wilcoxon signed-rank test was used. The mean number of intraoperative washes and wipes of the videoscope lens was 12 in the 10 cases before we used our method and 7.6 in the 5 cases after we used the novel technique, a difference that was significant (*P* = 0.0312).

## COMMENT

During UVATS, the energy device in one hand and suction device in the other hand are mainly used. Therefore, dissecting the sheath of vessels is sometimes difficult using counter traction as in conventional MVATS, because the suction cannula in the operator’s hand is used to maintain a good surgical field of view and to suction surgical smoke, mist and blood. Accordingly, if the operator’s nondominant hand holds the forceps, using the suction cannula is difficult because the assisting surgeon holds the thoracoscope and instruments such as the lung grasper or cotton swab to achieve adequate lung retraction in the dissection area. If the assisting surgeon retracts the lung using the suction cannula, the suction cannula aspirates the pleura of the lung, and surgical smoke cannot be ventilated.

It has been reported that using automatic smoke evacuation systems or anti-fogging agents is effective to reduce abdominal laparoscopic lens fogging [4, 5]. On the other hand, there are no reports on surgical smoke removal and prevention of lens fogging in UVATS. Our simple surgical smoke ventilation method does not interfere with either the surgeon’s visual field or the other instruments while providing good ventilation of surgical smoke, mist and moisture.

Moreover, since 2003, the introduction of the Diagnosis Procedure Combination payment system in Japan has mandated a reduction in medical costs for each patient. Although the suction catheter is disposable, it is inexpensive and cost effective (about 0.5 US dollars). In addition, anti-fogging solution can be omitted. We believe that our technique is effective for use during UVATS.

## ETHICS APPROVAL AND CONSENT TO PARTICIPATE

Ethics approval for this study was granted by the ethics committee of Hokkaido Cancer Center (approval number: 03-56).

## Funding

No funding was received.

**Conflict of interest:** none declared.

## Reviewer information

Interactive CardioVascular and Thoracic Surgery thanks Diego Gonzalez-Rivas and the other, anonymous reviewer(s) for their contribution to the peer review process of this article.
